# Augmented Mechanical Forces of the Surface-Modified Nanoporous Acupuncture Needles Elicit Enhanced Analgesic Effects

**DOI:** 10.3389/fnins.2019.00652

**Published:** 2019-06-20

**Authors:** Sun-Jeong Bae, Junsik Lim, Sangmin Lee, Hansaem Choi, Jae-Hwan Jang, Yu-Kang Kim, Ju-Young Oh, Jeong Hun Park, Hyuk-Sang Jung, Younbyung Chae, Su-Il In, Hi-Joon Park

**Affiliations:** ^1^Acupuncture and Meridian Science Research Center, Kyung Hee University, Seoul, South Korea; ^2^College of Korean Medicine, Kyung Hee University, Seoul, South Korea; ^3^College of Korean Medicine, Semyung University, Jecheon, South Korea; ^4^College of Korean Medicine, Dongguk University, Goyang, South Korea; ^5^Department of Energy Science and Engineering, DGIST, Daegu, South Korea; ^6^Graduate School of Korean Medicine, Kyung Hee University, Seoul, South Korea

**Keywords:** acupuncture analgesia, nanoporous needle, muscle, connective tissue, torque, complete Freund’s adjuvant

## Abstract

Over the past several decades, clinical studies have shown significant analgesic effects of acupuncture. The efficacy of acupuncture treatment has improved with the recent development of nanoporous needles (PN), which are produced by modifying the needle surface using nanotechnology. Herein, we showed that PN at acupoint ST36 produces prolonged analgesic effects in an inflammatory pain model; the analgesic effects of PN acupuncture were sustained over 2 h, while those using a conventional needle (CN) lasted only 30 min. In addition, the PN showed greater therapeutic effects than CN after 10 acupuncture treatments once per day for 10 days. We explored how the porous surface of the PN contributes to changes in local tissue, which may in turn result in enhanced analgesic effects. We showed that the PN has greater rotational torque and pulling force than the CN, particularly at acupoints ST36 and LI11, situated on thick muscle layers. Additionally, in *ex vivo* experiments, the PN showed greater winding of subcutaneous connective tissues and muscle layers. Our results suggest that local mechanical forces are augmented by the PN and its nanoporous surface, contributing to the enhanced and prolonged analgesic effects of PN acupuncture.

## Introduction

Acupuncture treatment has been used as a therapeutic modality in East Asia since 6000 BC, starting with a sharpened stone and progressing to stainless steel needles (White and Ernst, 2004; Wang et al., 2008). Acupuncture is of continuing global interest; the World Health Organization stated that acupuncture can be used to treat 63 diseases. Controlled clinical studies have shown its effectiveness in treating 28 diseases, including depression, nausea, vomiting, insomnia, and pain (Lao, 1996; Leake and Broderick, 1999). Among these conditions, treating pain is one of the most common uses of acupuncture therapy (Wang et al., 2008; Han, 2011; Vickers et al., 2012; Lee et al., 2013), particularly for treating lower back pain (Gunn et al., 1980; Macdonald et al., 1983; Manheimer et al., 2005; Lee et al., 2013) and osteoarthritis (Casimiro et al., 2005; Kwon et al., 2006).

Acupuncture is the practice of inserting needles into specific areas of the body surface, called acupoints, by penetrating the epidermis, dermis, subcutaneous layer, and/or muscle (Kimura et al., 1992; Park et al., 2011; Zhang et al., 2015). To enhance its therapeutic effect, acupuncture is often accompanied by manipulation of the needle involving rotation, vibration, and in-and-out movements (Stux et al., 2003; Meridians and Acupoints Compilation Committee of Korean Oriental Medical Colleges, 2015). Although details of the underlying mechanisms remain unclear, the impact of acupuncture is believed to involve various molecules [e.g., adenosine (Fredholm, 2007; Goldman et al., 2010), pERK (Park et al., 2014), and/or TRPV1 (Abraham et al., 2011; Wu et al., 2014; Liao et al., 2017)] and structures [e.g., subcutaneous connective tissue (Shen et al., 1973; Langevin et al., 2001b, 2002; Zhao, 2008), muscle (Shen et al., 1973), nerve (Kim et al., 2013), vessels (Huang T. et al., 2013), fibroblasts (Langevin et al., 2007, 2011), mast cells (Zhang et al., 2007; Fung, 2009), and/or keratinocytes (Geoffrey, 2009; Park et al., 2014)] near the acupoints, resulting in reconstitution and activation of various signals (Lin and Chen, 2008; Zhao, 2008; Park et al., 2014; Shu et al., 2016).

Because local changes induced by needle insertion and/or manipulation initiate the therapeutic effects of acupuncture, we hypothesized that potentiating the intensity of acupuncture stimulation could enhance its therapeutic efficacy. Thus, we developed a novel acupuncture needle, the micro/nanoporous acupuncture needle (PN). Unlike the smooth surface of a conventional acupuncture needle (CN), the PN surface has micro/nano-sized holes that provide a 20-fold increase in surface area. Previous studies have explored the properties of the PN surface produced using electrochemical techniques (Lee et al., 2017). Acupuncture using the PN showed a greater therapeutic effect than did the CN in an addiction model (In et al., 2016) and a colorectal cancer model (Lee et al., 2017). Other than the electrical conductivity (Not understood; how is porosity related to electrical conductivity?), the micro/nanoporous surface of PN has different physical characteristics that affect the therapeutic efficacy of acupuncture treatment. However, no studies have explored the physical impact of PN acupuncture, including the pullout force, mechanical load, and structural deformation induced by surface modification of local tissues during PN acupuncture.

In this report, we (i) analyzed various aspects of PNs and CNs on four different acupoints by measuring the pullout force and rotational torque using an Acusensor; (ii) compared the morphological deformation of local tissues induced by PN and CN acupuncture; and (iii) investigated changes in therapeutic effects induced by PN acupuncture by assessing the analgesic effects in an inflammatory pain model.

## Materials and Methods

### Experimental Animals

All experiments were performed using male C57BL/6 mice aged 7–10 weeks (Samtaco, Seoul, Korea). The mice were housed under 12-h light–dark cycle conditions with continuous access to chow and water. The mice were acclimated to the housing facility for 1 week before the experiments. All experiments were approved by the Dongguk University Animal Care Committee for Animal Welfare, and were performed according to the guidelines of the National Institutes of Health and the Korean Academy of Medical Sciences (IACUC-2017-022-1).

### Preparation of the PNs

To manufacture the PNs, conventional stainless steel acupuncture needles (8-mm length and 0.30-mm diameter; Dong Bang Acupuncture Inc., Boryeoung, South Korea) were anodized. First, the needles were washed sequentially with acetone, ethanol, and deionized (DI) water. Then, the stainless steel needles were anodized using a two-electrode cell. The cell was composed of a needle for the working electrode, carbon paper (2 cm × 0.5 cm × 0.042 cm carbon and fuel cell; CNL Technology, Suwon, South Korea) for the counter electrode, electrolytes with 0.2 wt.% NH_4_F (98%; American Chemical Society reagent, Alfa Aesar; Thermo Fisher Scientific, Waltham, MA, United States), and 2.0 vol% DI water in ethylene glycol. The needles were anodized for 30 min with 20 V (Paulose et al., 2006). Then, the anodized needles were rinsed with acetone, ethanol, and DI water, and dried in a nitrogen gas stream.

### Characterization of the PNs

The morphology of the needle surface was evaluated using a high-resolution scanning electron microscope (S-4800; Hitachi, Tokyo, Japan) operated at 3 kV. EIS measurements were obtained using a Biologics SAS (Model VSP-1158; Biologics, Cary, NC, United States) three-electrode workstation with a platinum wire as the counter electrode, Ag/AgCl electrode as the reference electrode and the needles as the working electrodes. The system was operated using EC Lab software in the frequency range of 100 kHz to 200 MHz. The electrolyte consisted of a saline solution (0.9 g NaCl in 100 mL DI water) purchased from JW-Pharma (Seoul, South Korea) and was used without further modifications.

### Measurements of the Rotational Torque and Pulling Force

Mice were anesthetized by intraperitoneal (i.p.) injection of Zoletil/Rompun and acupuncture needles (CN and PN, 8 mm in length, 0.18 mm in diameter; Dongbang Co., South Korea) were inserted to a depth of 3 mm at the ST36, LI11, ST25 and BL23 acupoints; Figure 2b shows the acupoints used in this study. Acupuncture using CNs and PNs was performed as follows: (1) without manipulation, or (2) with manipulation (consisting of clockwise rotations of 360°/s for nine spins). Pulling and rotational forces of the acupuncture needling were measured using the Acusensor (Stromatec, Inc., Burlington, VT, United States) motion sensor system (Figure 2a). The vertical force acting on the needle to resist its insertion and pulling, and the rotational force acting on the needle to resist its rotation, were measured using a motion sensor. The data of each acupoint were analyzed respectively. And overall values were also calculated using average data.

### Histochemical Staining of Abdominal Tissues

#### Sampling

We performed histochemical analyses of the abdominal tissues around the needle. The large surface area and lack of bones in the abdominal tissues allowed for rapid histological analyses of acupuncture treatment (Langevin et al., 2001a). Mice were anesthetized by Zoletil/Rompun (i.p.). The CNs and PNs (or NN) were inserted on both sides of the abdomen in a random order. Then, the needles were manipulated by nine unilateral spins, with one spin consisting of a clockwise rotation of 360°. For all insertions, the needle depth was held constant at 3 mm, with the needle penetrating the entire abdominal wall, and the track was labeled with tissue-marking dye (Davidson Marking System, Bradley Products, Inc., Bloomington, MN, United States). After the manipulation, the mice were sacrificed without removing the needle, and the whole body was immersion-fixed in 10% formalin for 48 h. Then, the abdominal tissues were excised, including the epidermis to the abdominal muscle layer and the needle, and fixed again in 10% formalin overnight.

#### Histological Analysis

The needles were removed after paraffinization of the samples and before paraffin embedding. Tissue blocks were cut parallel to the needle axis at 8-μm thickness using a manual rotary microtome (Shandon Finesse 325; Thermo Fisher Scientific Inc.). Every fifth slide with a needle track label was stained with hematoxylin (Merck Co., Darmstadt, Germany) and eosin (Sigma–Aldrich Co., St. Louis, MO, United States), or Masson trichrome-counterstained with aniline blue (Masson Trichrome Stain Kit, Nova Ultra^TM^; IHC World, Ellicott City, MD, United States), which stains collagen as blue and muscle as red. Then, the thicknesses of the epidermis, dermis, subcutaneous, and abdominal muscle layers along the needle track were measured using a light microscopy (BX51, Olympus, Japan), with the subcutaneous layer divided into adipose tissue, cutaneous muscle, and subcutaneous connective tissue.

### Behavioral Experiments

#### Experimental Groups and Acupuncture Treatment

Acupuncture was performed at the ST36 (Joksamli or Zusanli) acupoint, located 3–4 mm below and 1–2 mm lateral to the midline of the knee (Lim, 2010) (Figure 2b). ST36 is one of the most effective points in East Asian medicine and its anti-nociceptive effects in rodent CFA models of chronic pain are well-established (Goldman et al., 2010; Wu et al., 2014; Lu et al., 2016).

The mice were divided into five treatment groups: treatment with CFA only (CFA), treatment with CFA and non-invasive acupuncture (NN) needling at the ST36 acupoint (CFA+NN), treatment with CFA and CN at the ST36 acupoint (CFA+CN), treatment with CFA and PN at the ST36 acupoint (CFA+PN), and control (CON) (*n* = 7–8 in each group).

The mice in the CFA+CN and CFA+PN groups were immobilized by hand, and the needles were inserted to a depth of 3 mm at the bilateral acupoints. Then, the acupuncture needles were turned at a rate of two spins/s bidirectionally (one spin consisted of clockwise rotation of 180° and a counterclockwise rotation of 180°) for 30 s. The needles were removed immediately after manipulation. To create a blunt non-invasive acupuncture needle, the sharp end of the acupuncture needle was snipped. The mice in the CFA+NN group were pressed with 50 mN of force non-invasively, and no manipulation was done for 30 s. The mice in the CON and CFA groups were also immobilized by hand for 1 min to ensure an equal amount of stress among the acupuncture groups. The treatments began at 4 days after CFA injection once per day for 10 days. Figure 7A,B shows the schedule of the acupuncture treatments and behavioral experiments.

#### CFA-Induced Mouse Pain Model

CFA oil suspension was diluted 1:1 with saline and used to induce chronic inflammation. The mice were anesthetized with ether before the injection. The mice in the CFA, CFA+NN, CFA+CN, and CFA+PN groups received a subcutaneous injection of 100 μL CFA emulsion solution into the plantar of both hind paws, and the mice in the CON group received identical injections with equal amounts of saline. CFA-treated mice developed signs of inflammation that were prominent at 4 days after the injection.

#### Mechanical Threshold Assessment

Mechanical allodynia was assessed by testing the withdrawal response to von Frey filaments. Before the baseline test, the mice were habituated in 8 × 10 × 10 cm acrylic boxes with gridded floors, daily for 1 h for 2 days. Mechanical thresholds were assessed before CFA injection, as well as and after acupuncture treatment (Figure 7A). Prior to each test, the mice were habituated again for 1 h. The mechanical allodynia of the hind paws was assessed using an electronic von Frey filament (Figure 7B). The filament was applied to the plantar surface of the hind paw with 0.6 g of force for 1 s. All mice underwent 10 applications on both hind paws, with a 5 s interval between each application. Frequency was defined according to positive responses from 10 applications. The assessor was blinded to the group assignment, and an experienced investigator who was completely blinded counted the withdrawal frequencies.

To observe the short-term changes of allodynia induced by acupuncture, tests were performed before an acupuncture treatment and every 30 min for 2 h after the treatment (0, 30, 60, 90 and 120 min after acupuncture treatment). We tested these short-term effects only after the 1st and the 8th acupuncture treatment, to minimize the stress. And to test the long-term changes, we tested mechanical allodynia on the 1st, 3rd, 7th, and 10th day of acupuncture treatment (Figure 7A).

### Statistical Analyses

GraphPad Prism 7 software (GraphPad Software Inc., La Jolla, CA, United States) was used for the statistical analyses. All data are expressed as means and standard error of the mean (SEM). Statistical analyses were performed using one-way analysis of variance (ANOVA), two-way ANOVA or repeated measures of ANOVA, where appropriate. The Bonferroni *post hoc* test was followed. In all analyses, *p* < 0.05 was considered to indicate significant differences.

## Results

### Physical Properties of PNs

**FIGURE 1:**
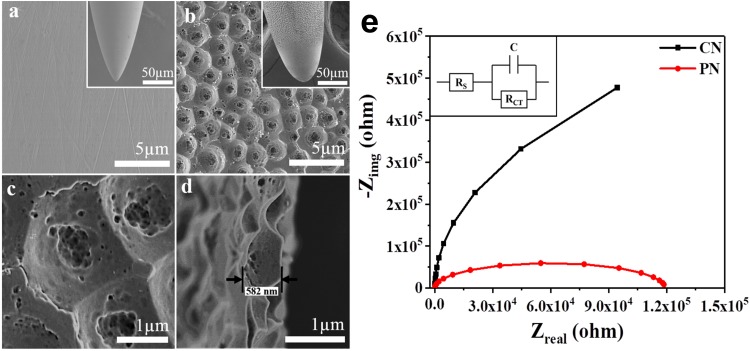
Surfaces of the conventional needle (CN) **(a)** and micro-nanoporous needle (PN) **(b)**. High-resolution image of the PN acupuncture needle **(c)** and cross-sectional image of the PN anodized needle **(d)**. Electrochemical impedance spectra (EIS) with fitted Nyquist plots corresponding to the CN and PN **(e)**. The inset shows equivalent circuits for EIS measurements, where RS = solution resistance, RCT = charge transfer resistance, and C = double layer capacitance.

Figure 1a–d shows field emission scanning electron microscopy (FE-SEM) images of stainless steel CNs compared with PNs, fabricated by anodizing CNs using an electrolyte of 0.2 wt.% NH_4_F and 2 vol.% DI water in ethylene glycol at room temperature for 30 min at 20 V (Paulose et al., 2006). Initially, the CN had a smooth surface (Figure 1a); after anodization, the needle showed a micro-nanoscale porous surface topology with a high surface area (Figure 1b). High-resolution images of the PN showed uniform formation of conical-shaped pores (Figure 1c); a cross-sectional image of the porous topology is shown in Figure 1d.

Electrochemical impedance spectroscopy (EIS), a powerful technique for characterizing surfaces (Ehsani et al., 2014), was used to investigate CNs and PNs. Figure 1e shows the fitted Nyquist plots corresponding to the EIS results for CNs and PNs immersed in saline solution. The Nyquist plots for both needles show similar shapes, indicating that saline had no effect on the needle surface. Depressed semicircles are formed due to the combination of the charge transfer resistance (*R*_CT_) and the constant phase element of the working electrode (acupuncture needles) and the electrolyte interface (Ehsani et al., 2013). A reasonable decrease was observed in the semicircle diameter in the PNs, indicative of a significant decrease in R_CT_.

### Measurements of Rotational Torque and Pullout Force at Four Representative Acupoints

**FIGURE 2:**
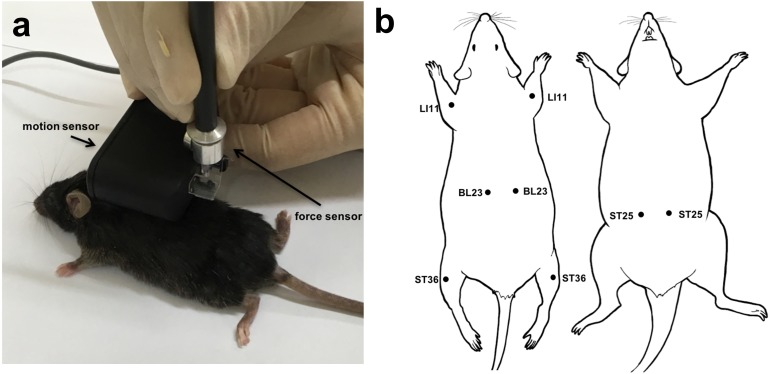
Image of acupuncture needling using the Acusensor **(a)**. Acupoints used in this study **(b)**.

The rotational torque and pullout force of PNs and CNs were measured using the Acusensor motion sensor system (Figure 2a). Four representative acupoints at the mouse hindlimb (ST36), forelimb (LI11), abdomen (ST25), and back (BL23) (Figure 2b) were used to identify and compare mechanical loads between PN and CN at each acupoint.

**FIGURE 3:**
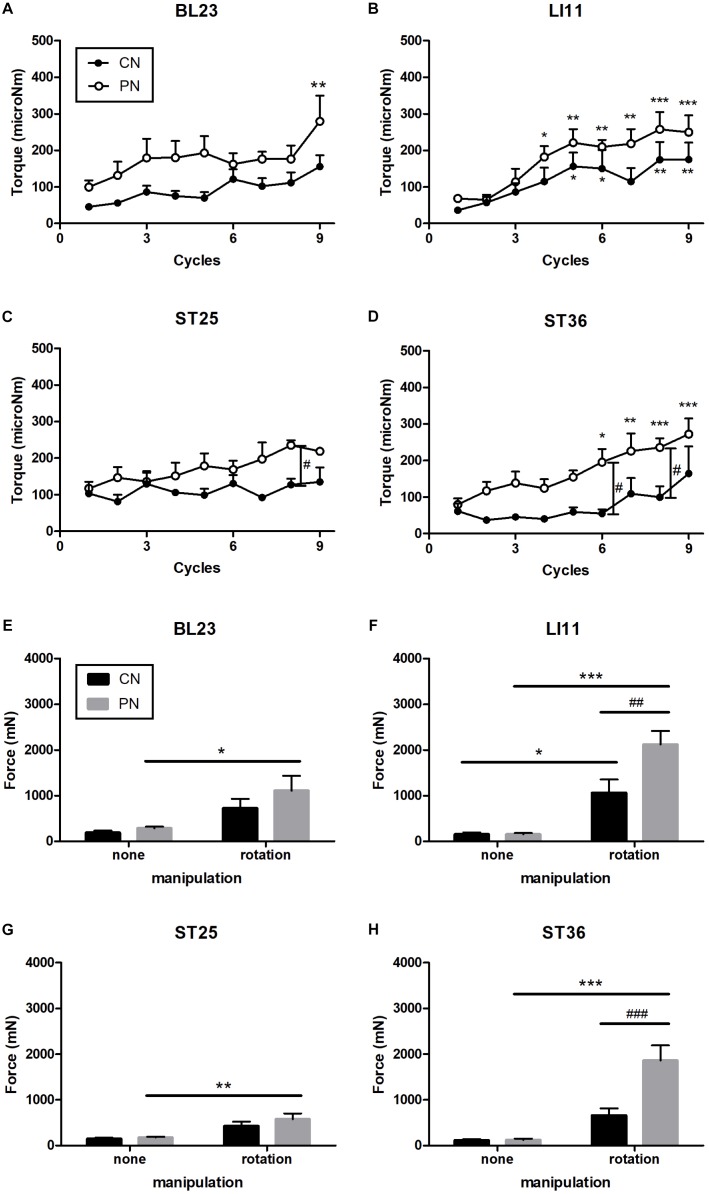
Measurement of rotational torque at acupoints ST36 **(A)**, LI11 **(B)**, ST25 **(C)**, and BL23 **(D)**. Measurement of pulling force at ST36 **(E)**, LI11 **(F)**, ST25 **(G)**, and BL23 **(H)**. Rotation of the PN significantly increased the pulling force. At ST36, the pulling force of the PN with rotation was significantly higher than the CN with rotation. With increased numbers of rotation cycles, the torque in the PN and CN increased. In addition, PN had significantly stronger torque than did CN at ST25 and ST36 (^∗^*P* < 0.05, ^∗∗^*P* < 0.01, ^∗∗∗^*P* < 0.001, compared to the no-manipulation group; at the 1st cycle: ^#^*P* < 0.05, ^##^*P* < 0.01, ^###^*P* < 0.001, CN vs. PN; Two-way repeated-measures ANOVA **(A–D)** and two-way ANOVA **(E–H)** followed by the Bonferroni test; each *n* = 4–9). Error bars indicate the standard error of the mean (SEM).

The rotational torque was measured during nine rotations at each acupoint (Figure 3A–D). The torque induced by PN and CN was enhanced as the number of rotations was increased in both PN and CN groups, and the rate of increase was much higher in the PN than in the CN group. Especially, the PN group showed significantly higher torque than the CN group when acupuncture was administered to ST36 (*P* < 0.05 at both the 6th and 8th rotation) and to ST25 (*P* < 0.05 at the 8th rotation).

The pullout force was measured after nine rotations of the needles (Figure 3E–H). These rotations induced trends of increased pullout force compared to no rotations for both the PN and CN groups; the increase was significant at acupoint ST36 (only PN: *P* < 0.001), LI11 (CN: *p* < 0.05, PN: *P* < 0.001), ST25 (only PN: *P* < 0.05), and BL23 (only PN: *P* < 0.01). As shown in Figure 3E–H, compared with the CN group, the PN group showed significantly higher pullout force at ST36 (*P* < 0.001) and LI11 (*P* < 0.01). No increase in pullout force was found for either needle group when the needle was inserted without rotation.

**FIGURE 4:**
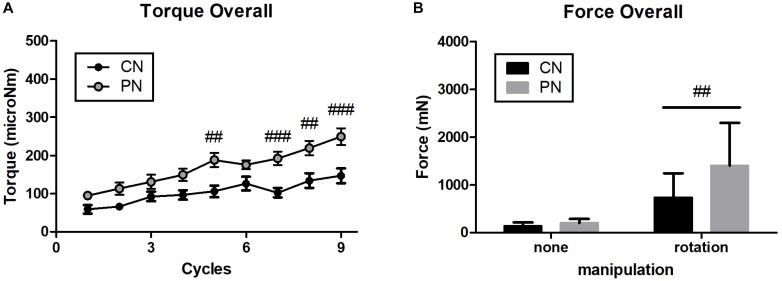
Overall value of rotational torque **(A)** and pulling force **(B)**. PNs had significantly stronger pulling force and rotational torque than CNs (^##^*P* < 0.01, ^###^*P* < 0.001, CN vs. PN; two-way ANOVA followed by the Bonferroni test; each *n* = 16). Error bars indicate the SEM.

To explore differences in mechanical load between PNs and CNs on local tissues, we calculated the overall pulling force and rotational torque by averaging data of each location (Figure 4). The results showed that the overall rotational torque of the PN group increased to a more significant degree than did that of the CN group (*P* < 0.01, *P* < 0.001, *P* < 0.01, and *P* < 0.001 at the 5th, 7th, 8th, and 9th rotations, respectively; Figure 4A). The overall pulling force of the PN group was significantly higher than that of the CN group (*P* < 0.01, Figure 4A).

### Histological Analysis in Local Tissues After Acupuncture

**FIGURE 5:**
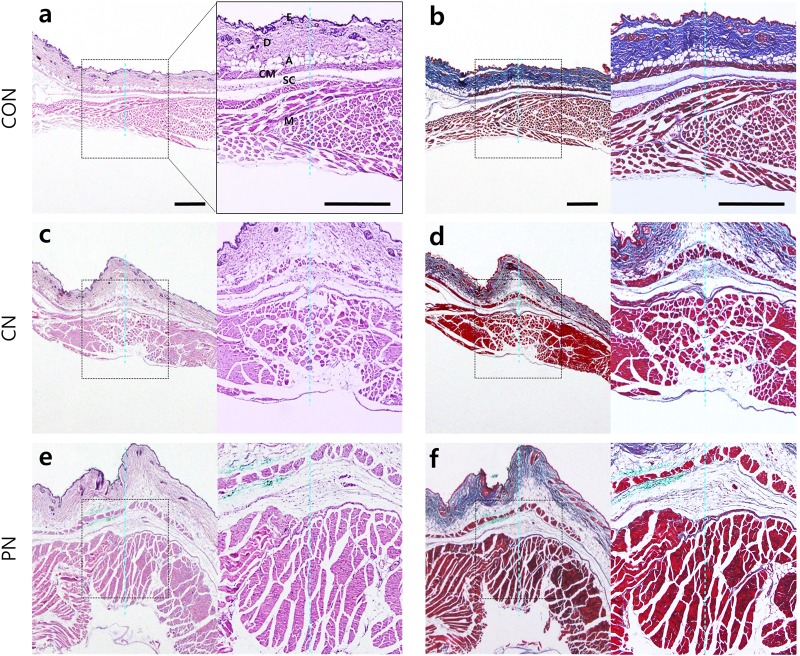
Histological images in abdominal wall tissues. Control [CON **(a,b)**, CN **(c,d)**, and PN **(e,f)**]. Histological analyses were performed after nine acupuncture rotations. The adjacent sections from the same tissues were stained with hematoxylin and eosin (H&E) **(a,c,e)** or Masson’s trichrome counterstained with aniline blue **(b,d,f)**. The histological layer includes the epidermis (E), dermis (D), adipose (A), cutaneous muscle (CM), subcutaneous connective tissue (SC), and abdominal muscle (M). The dotted blue line indicates the inserted trace of acupuncture needle. Scale bar, 500 μm.

**FIGURE 6:**
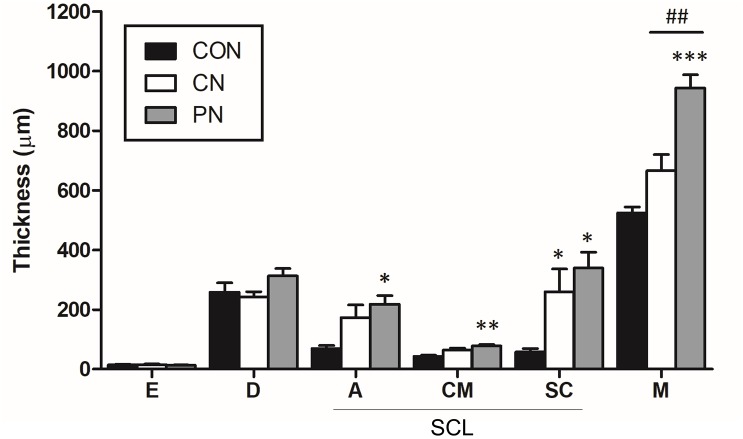
Thickness of each layer in the abdominal wall. The thickness of each tissue layer was measured at the epidermis (E), dermis (D), adipose (A), cutaneous muscle (CM), subcutaneous connective tissue (SC), and abdominal muscle (M). SCL represents the subcutaneous layer. ^∗^*P* < 0.05, ^∗∗∗^*P* < 0.001, compared to the CON group; one-way ANOVA followed by the Bonferroni test; each *n* = 4–5. Error bars indicate the SEM.

Histological analysis using hematoxylin and eosin staining (Figure 5a,c,e) or Masson’s trichrome staining (Figure 5b,d,f) was performed to investigate acupuncture-induced morphological changes in local tissues, including the epidermis, dermis, subcutaneous tissue (adipose, cutaneous muscle, and subcutaneous connective tissue), and muscle layers. Masson’s trichrome staining of the samples revealed that collagen-containing layers such as dermis, adipose, and subcutaneous connective tissue were stained blue; the epidermis, cutaneous muscle, and abdominal muscle were stained red. The measured thicknesses of the layers are shown in Figure 6.

Histological analysis showed that the thickness of subcutaneous layers increased after rotating CNs and PNs. In the adipose layer, both CNs and PNs increased the thickness, but only PNs showed a significant change compared to the CON group (CON: 69.4 ± 10.3 μm; CN: 173.6 ± 42.1 μm; PN: 218.5 ± 28.1 μm, *P* < 0.05 CON vs. PN). In the cutaneous muscle layer, both CNs and PNs increased the thickness, but only PNs induced a significant difference compared to the CON group (CON: 42.9 ± 4.3 μm; CN: 64.2 ± 7.0 μm; PN: 78.1 ± 4.1 μm, *P* < 0.01 vs. CON). In subcutaneous connective tissue, both CNs and PNs induced significantly greater thickness compared to the CON group (CON: 58.0 ± 11.7 μm; CN: 259.2 ± 77.0 μm; PN: 339.2 ± 53.3 μm, all *P* < 0.05 vs. CON); there was no difference between CNs and PNs (Figure 5, 6). Masson’s trichrome staining showed a whorl pattern in collagen fibers in subcutaneous connective tissues in the CN and PN groups (Figure 5b,d,f).

Based on muscle layer analysis, morphological changes in muscle thickness were only significant with PNs: the thickness using PNs (942.7 ± 44.8 μm) was significantly greater than that using CNs (665.4 ± 122.0 μm, PN vs. CN: *P* < 0.01) and that for the CON group (524.7 ± 38.3 μm, PN vs. CON: *P* < 0.001, Figure 5, 6). Masson’s trichrome staining analyses showed significant whorl patterns in muscles layers in the PN group (Figure 5b,d,f).

No morphological changes in the dermis and epidermis were observed in either group.

### Effects of PN Acupuncture on the Mechanical Pain Threshold in Complete Freund’s Adjuvant (CFA)-Induced Inflammatory Pain Model

**FIGURE 7:**
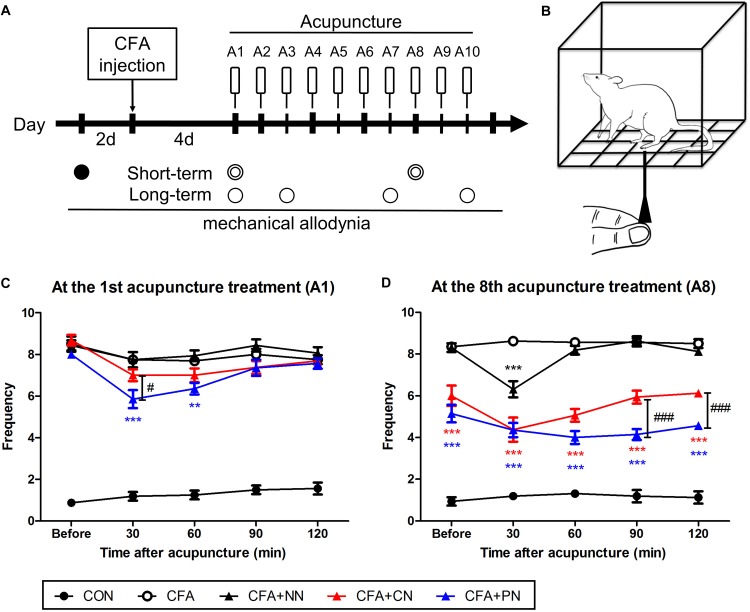
The short-term effects of porous and conventional acupuncture needles in the CFA-induced inflammatory pain model. Timeline of the mechanical threshold assessment in the complete Freund’s adjuvant (CFA)-induced pain model **(A)** Starting 4 days after CFA treatment, acupuncture treatment was administered once per day for 10 days (A1–A10, total 10 times). The Von Frey test was conducted as follows: at 2 days before CFA injection (∙, baseline); at 30, 60, 90, and 120 min after acupuncture treatment (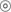); and at 1 day after acupuncture (∘). Schematic diagram of the von Frey test **(B)**. The analgesic effect of acupuncture under the CFA-induced inflammatory pain model was assessed after the 1st **(C)** and 7th **(D)** acupuncture treatments (^∗∗^*P* < 0.01, ^∗∗∗^*P* < 0.001, compared to the CFA; ^#^*P* < 0.05, ^###^*P* < 0.001, CN vs. PN; two-way repeated measures ANOVA followed by the Bonferroni test; each *n* = 7–8) **(C,D)**. Error bars indicate the SEM.

We investigated the short-term and long-term analgesic effects of CN and PN acupuncture in a CFA-induced inflammatory pain model. Acupuncture was performed at acupoint ST36 (Figure 2b), and mechanical allodynia was measured using Von Frey filaments (Figure 7B). The baseline mechanical threshold before CFA injection was similar across all groups. Acupuncture treatments with CN and PN started 4 days after CFA injection, when the pain reached a stable level.

At the 1st acupuncture treatment, both CN and PN attenuated mechanical allodynia at 30–60 min post-acupuncture; however, only the PN group (5.9 ± 0.4) showed a significant difference compared to the CFA group (7.8 ± 0.4, *P* < 0.001) at 30 min post-acupuncture. Moreover, allodynia scores of the PN group were significantly lower than those of the CN group (7.0 ± 0.3, *P* < 0.05) at the same time point. At 120 min after treatment, mechanical allodynia in both the CN (7.69 ± 0.2) and PN (7.57 ± 0.3) groups returned to the pre-treatment values (Figure 7A).

After the 7th acupuncture treatment, the short-term effects of PN and CN acupuncture were tested again using the same protocol. As previously shown (Zhao, 2008; Goldman et al., 2010), the most effective time for CN acupuncture was 30 min after acupuncture (4.4 ± 0.6, *P* < 0.001 vs. CFA group), with the effect returning to baseline after 90 min. In contrast, in the PN group, the level of mechanical allodynia decreased gradually until 90 min after acupuncture (4.0 ± 0.3, *P* < 0.001 vs. CFA group), and the decrease continued until 120 min after acupuncture.

A comparison of the pain levels of those in the PN group with that of those in the CN group showed that the PN group exhibited significantly less pain than did the CN group at 90 and 120 min after acupuncture (both *P* < 0.001, Figure 7B).

**FIGURE 8:**
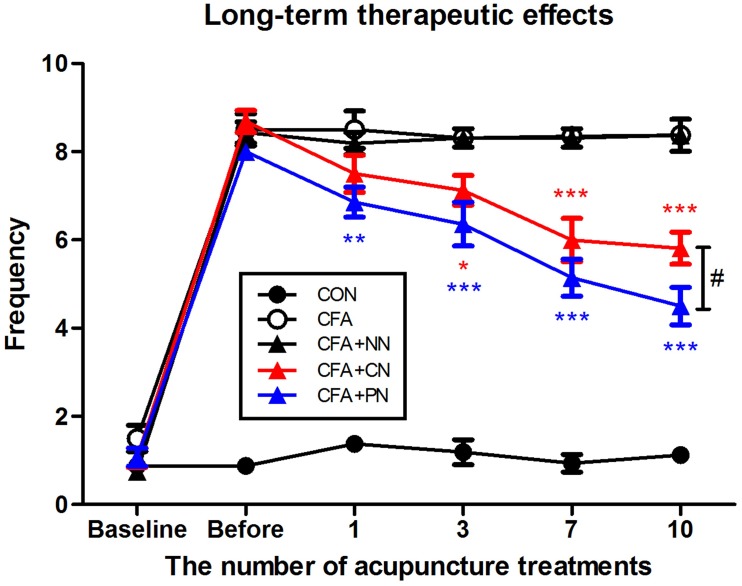
Therapeutic effects of porous and conventional acupuncture needles in the CFA-induced inflammatory pain model. The paw withdrawal frequency of each mouse was measured 24 h after acupuncture treatments. The PN group showed a greater therapeutic effect than the CN group (^∗^*P* < 0.05, ^∗∗^*P* < 0.01, ^∗∗∗^*P* < 0.001, compared to the CFA group; ^#^*P* < 0.05, CN vs. PN; two-way repeated measures ANOVA followed by the Bonferroni test; each *n* = 7–8). Error bars indicate the SEM.

In addition to the short-term analgesic effect, we observed long-term therapeutic effects measured 24 h after each acupuncture treatment. Throughout 10 acupuncture treatments (once per day for 10 days), both the PN and CN groups showed gradual alleviation of mechanical allodynia (PN vs. CFA, *P* < 0.01 after the 1st acupuncture session; CN vs. CFA, *P* < 0.05 after the 2nd acupuncture session). The difference between the PN and CN groups gradually increased as the number of treatments increased; the anti-allodynic effect of PN (4.5 ± 0.4) was significantly greater than that of CN (5.8 ± 0.4) after 10 acupuncture sessions (measured 1 day after the 10th acupuncture treatment, *P* < 0.05, Figure 8).

## Discussion

Although PNs have been developed and applied for improving acupuncture treatment efficacy (In et al., 2016; Lee et al., 2017; Sorcar et al., 2018), no study has explored their analgesic effect, mechanical load, or influence on tissues. Therefore, we investigated the local impact and morphological changes caused by PN acupuncture and measured the difference in analgesic effects between PN and CN acupuncture. The results are summarized as follows: (1) PN induced larger mechanical load than CN; (2) likewise, the PN treatment tended to produce greater morphological changes in muscle layer thickness around the needle track than the CN treatment; and (3) in the CFA inflammatory pain mouse model, PN acupuncture at ST36 resulted in a longer duration of analgesic effect and longer-term treatment efficacy compared with CN acupuncture.

The different impact of PN on local tissues compared with that of CN is likely due to the high surface area of the needle. In et al. reported that the surface area of a PN is 20× greater than that of a CN of the same diameter (In et al., 2016). FE-SEM images confirm the presence of micro-nano porosity on the PN surface. This porosity significantly increases the PNs contact with the surrounding environment; the smaller R_CT_ of EIS implies a greater surface area (Ehsani et al., 2013, 2014). The larger surface area enhances the manipulation-induced frictional force between the needle and adjacent tissues, in turn enhancing the needle-grasp force (Kwon et al., 2017). PNs showed greater deformation of tissues (subcutaneous and muscle layers) around the needle and higher needle-grasp compared to CNs, as measured by the pulling force and torque on four different acupoints.

Histological examination of mouse abdominal tissues indicated that PN acupuncture significantly thickened the subcutaneous connective tissues and muscle layer, resulting in thicker layers than those induced by CN acupuncture, especially in the muscle. Subcutaneous connective tissue and muscle are the two main components of needle grasp, facilitating the therapeutic effect (Shen et al., 1973; Langevin et al., 2002; Zhao, 2008). Rotational manipulation of the acupuncture needle facilitates the gathering of collagen fibers of connective tissues around the needle (Langevin et al., 2006), transmitting mechanical stimulation to the surrounding cells and activating a cellular response in the fibroblasts of connective tissues (Burridge et al., 1988; Banes et al., 1995). Muscle contraction is also believed to induce needle grasp, and its polymodal-type receptor in deep tissue is associated with needle grasp and therapeutic effects (Shen et al., 1973; Lin et al., 1979). Furthermore, other tissue layers (e.g., the epidermis and dermis) may play a role in the effects of acupuncture (Langevin et al., 2007, 1; Abraham et al., 2011; Park et al., 2014); however, morphological changes were minimal in this study.

The exact mechanism through which PNs exert a greater effect cannot be determined based on morphological observation. However, our results are consistent with previous reports suggesting that a greater dose of acupuncture stimulation leads to greater changes at the molecular level, increasing signal transduction and thus exerting an enhanced effect (Goldman et al., 2010; Zylka, 2010; Abraham et al., 2011; Park et al., 2014; Wu et al., 2014). This newly developed PN acupuncture method results in greater stimulation of tissues around the needle track and enhances spinal dorsal horn neuronal activity by acupuncture stimulation in rats (In et al., 2016). Several studies have shown that PNs enhance the therapeutic effect of acupuncture (In et al., 2016; Lee et al., 2017; Sorcar et al., 2018); however, this study is the first to describe the analgesic effect of PNs, the most representative effect of acupuncture.

This study showed that the short-term analgesic effect of PN acupuncture at ST36 was greater than that of CN acupuncture in the CFA-induced pain mouse model. PN acupuncture showed greater immediate (30 min after treatment) and prolonged (90–120 min after treatment) analgesic effects. Our results are in agreement with studies showing higher efficacy and greater therapeutic effects of PN acupuncture in an addiction model (In et al., 2016) and a colorectal cancer model (Lee et al., 2017) compared with CN acupuncture.

Morphologically, CN acupuncture needle rotations induce collagen winding in subcutaneous connective tissues with no structural changes in the muscle layers (Langevin et al., 2001a). In contrast, PN acupuncture resulted in winding of both muscle and subcutaneous connective tissues. The difference in needle grasp between the PN and CN was noticeable at ST36 and LI11. Anatomically, ST36 and LI11 are situated on thicker muscle layers (i.e., the tibialis anterior muscle for ST36 and extensor carpi radialis longus muscle for LI11) compared to ST25 and BL23. In the upper skin layer, the collagen fibers are relatively small, and only needle tracks can be observed rather than tissue twirling; thus, morphological changes appear to be relatively minimal. However, the lower degree of morphological change does not indicate that there are no biological changes associated with the therapeutic effects of acupuncture. Park et al. reported that acupuncture manipulation increased the expression level of pERK in keratinocytes of the epidermis, which may be associated with the analgesic effects of acupuncture. Further studies should be performed simultaneously considering both morphological and molecular changes. Although our results do not preclude the participation of other layers in the acupuncture effects, this study supports that muscle layers may play an important role in the improvements induced by PNs. Taken together, these findings suggest that both subcutaneous connective tissue and muscle contribute to the effects of acupuncture; however, the enhanced therapeutic effects by PNs may be associated with the effects on muscle layers.

Changes in subcutaneous connective tissue in the CN group were less marked than those reported previously (Li et al., 2005; Langevin et al., 2011; Huang C.-P. et al., 2013; Lu et al., 2016). This difference is likely due to the less marked manipulation in the present versus previous studies (Langevin et al., 2002, 2007) because a smaller manipulation using PNs was sufficient to produce an effective change similar to that reported previously (Kwon et al., 2017).

Our study has several limitations. First, to perform the experiments more precisely, sample size calculation is recommended based on a previous study (Festing and Wilkinson, 2007; Kar and Ramalingam, 2013). Since no previous studies have evaluated the analgesic effects of PN and CN, we could not calculate the sample size for this experiment. For subsequent large-scale studies of CN and PN groups, the sample size was calculated using G^∗^Power (G^∗^Power 3.1.9.2 for Windows 10, Faul et al., 2007).^1^ The measurement of mechanical pain in the CN and PN groups was used as ground data. The sample size was calculated using the independent *t*-test with an α-level of 0.05 and power of 80%. The number calculated was 10 per group, with an effect size d:1.219424, critical *t*: 1.7340636, Df: 18, non-centrality parameter δ:2.7267150, and actual power: 0.8357533. Next, our results did not reveal whether the PN had better effects than the CN at the other acupoints (excluding ST36). Therefore, further studies are required to compare the effects of the two types of needles using clinically relevant disease models with each acupoint. For example, the effects of ST25 can be tested in a visceral pain or colitis model (Shi et al., 2011; Zhu L. et al., 2018; Zhu X. et al., 2018; Ji et al., 2019). Differences in structural changes according to the modified needle surfaces may be associated with greater analgesic efficacy, but the detailed mechanisms should be explored. In addition, further experiments are required to characterize the enhanced analgesic effect of PN and its detailed mechanism in humans.

Overall, this study is the first to report that the PN, with its large surface area, shows a greater needle grasping power than the CN, thus producing greater morphological changes in subcutaneous and muscle tissues. More importantly, we showed that this treatment increases the analgesic effects with prolonged time. This study suggests that the PN is an effective tool for improving acupuncture efficacy in clinical medicine.

## Ethics Statement

All experiments were approved by the Dongguk University Animal Care Committee for Animal Welfare, and were performed according to the guidelines of the National Institutes of Health and the Korean Academy of Medical Sciences (IACUC-2017-022-1).

## Author Contributions

JP, S-JB, JL, and SL conceived and designed the study. S-II, J-HJ, S-JB, JL, SL, and YC developed the methodology. SB, JL, SL, Y-KK, and HC acquired the data. S-JB, JL, SL, H-SJ, and J-YO analyzed and interpreted the data. SB, JL, SL, H-JP, and S-II wrote, reviewed, and revised the manuscript. H-JP, S-II, and JP administrated the technical or material support. H-JP supervised the study. S-JB, JL, and SL drew the Figure 2, 7. All authors had input into the manuscript and have approved the manuscript for publication.

## Conflict of Interest Statement

The authors declare that the research was conducted in the absence of any commercial or financial relationships that could be construed as a potential conflict of interest. The handling Editor is currently organizing a Research Topic with one of the authors, YC, and confirms the absence of any other collaboration.

## Footnotes

1 http://www.gpower.hhu.de

## References

[B1] Abraham T. S. Chen M. L. Ma S. X. (2011). TRPV1 expression in acupuncture points: response to electroacupuncture stimulation. *J. Chem. Neuroanat.* 41 129–136. 10.1016/j.jchemneu.2011.01.001 21256210 PMC3117662

[B2] Banes A. J. Tsuzaki M. Yamamoto J. Brigman B. Fischer T. Brown T. (1995). Mechanoreception at the cellular level: the detection, interpretation, and diversity of responses to mechanical signals. *Biochem. Cell Biol.* 73 349–365. 10.1139/o95-043 8703408

[B3] Burridge K. Fath K. Kelly T. Nuckolls G. Turner C. (1988). Focal adhesions: transmembrane junctions between the extracellular matrix and the cytoskeleton. *Annu. Rev. Cell Biol.* 4 487–525. 10.1146/annurev.cb.04.110188.002415 3058164

[B4] Casimiro L. Barnsley L. Brosseau L. Milne S. Welch V. Tugwell P. (2005). Acupuncture and electroacupuncture for the treatment of rheumatoid arthritis. *Cochrane Database Syst. Rev.* 19:CD003788. 10.1002/14651858.CD003788.pub2 16235342 PMC8729824

[B5] Ehsani A. Jaleh B. Nasrollahzadeh M. (2014). Electrochemical properties and electrocatalytic activity of conducting polymer/copper nanoparticles supported on reduced graphene oxide composite. *J. Power Sources* 257 300–307. 10.1016/j.jpowsour.2014.02.010

[B6] Ehsani A. Mahjani M. G. Bordbar M. Adeli S. (2013). Electrochemical study of anomalous diffusion and fractal dimension in poly ortho aminophenol electroactive film: comparative study. *J. Electroanal. Chem.* 710 29–35. 10.1016/j.jelechem.2013.01.008

[B7] Faul F. Erdfelder E. Lang A.-G. Buchner A. (2007). G^∗^Power 3: a flexible statistical power analysis program for the social, behavioral, and biomedical sciences. *Behav. Res. Methods* 39 175–191. 10.3758/BF03193146 17695343

[B8] Festing S. Wilkinson R. (2007). The ethics of animal research. Talking point on the use of animals in scientific research. *EMBO Rep.* 8 526–530. 10.1038/sj.embor.7400993 17545991 PMC2002542

[B9] Fredholm B. B. (2007). Adenosine, an endogenous distress signal, modulates tissue damage and repair. *Cell Death Differ.* 14 1315–1323. 10.1038/sj.cdd.4402132 17396131

[B10] Fung P. C. W. (2009). Probing the mystery of Chinese medicine meridian channels with special emphasis on the connective tissue interstitial fluid system, mechanotransduction, cells durotaxis and mast cell degranulation. *Chin. Med.* 4:10. 10.1186/1749-8546-4-10 19480699 PMC2694206

[B11] Geoffrey B. (2009). Acupuncture: a novel hypothesis for the involvement of purinergic signalling. *Med. Hypotheses* 73 470–472. 10.1016/j.mehy.2009.05.031 19628336

[B12] Goldman N. Chen M. Fujita T. Xu Q. Peng W. Liu W. (2010). Adenosine A1 receptors mediate local anti-nociceptive effects of acupuncture. *Nat. Neurosci.* 13 883–888. 10.1038/nn.2562 20512135 PMC3467968

[B13] Gunn C. C. Milbrandt W. E. Little A. S. Mason K. E. (1980). Dry needling of muscle motor points for chronic low-back pain: a randomized clinical trial with long-term follow-up. *Spine* 5 279–291. 6446774 10.1097/00007632-198005000-00011

[B14] Han J. S. (2011). Acupuncture analgesia: areas of consensus and controversy. *Pain* 152 S41–S48. 10.1016/j.pain.2010.10.012 21078546

[B15] Huang C.-P. Chen H.-N. Su H.-L. Hsieh C.-L. Chen W.-H. Lai Z.-R. (2013). Electroacupuncture reduces carrageenan- and CFA-induced inflammatory pain accompanied by changing the expression of Nav1.7 and Nav1.8, rather than Nav1.9, in mice dorsal root ganglia. *Evid. Based Compliment. Alernat. Med.* 2013:312184. 10.1155/2013/312184 23573123 PMC3615619

[B16] Huang T. Yang L. Jia S. Mu X. Wu M. Ye H. (2013). Capillary blood flow in patients with dysmenorrhea treated with acupuncture. *J. Trad. Chin. Med.* 33 757–760. 10.1016/S0254-6272(14)60008-X 24660607

[B17] In S. Gwak Y. S. Kim H. R. Razzaq A. Lee K.-S. Kim H. Y. (2016). Hierarchical micro/nano-porous acupuncture needles offering enhanced therapeutic properties. *Sci. Rep.* 6:34061. 10.1038/srep34061 27713547 PMC5054419

[B18] Ji M. X. Guo M. W. Gao Y. S. Lan Y. Wang S. Wang Y. F. (2019). Comparison of effects of electroacupuncture at “Tianshu” (ST25) and “Dachangshu” (BL25) on intestinal sensitivity and expression of muscarinic M3R and 5-HT3AR in irritable bowel syndrome rats. *Zhen Ci Yan Jiu* 44 264–269. 10.13702/j.1000-0607.180764 31056879

[B19] Kar S. S. Ramalingam A. (2013). Is 30 the magic number? Issues in sample size estimation. *Nat. J. Commun. Med.* 4 175–179.

[B20] Kim S. A. Lee B. H. Bae J. H. Kim K. J. Steffensen S. C. Ryu Y.-H. (2013). Peripheral afferent mechanisms underlying acupuncture inhibition of cocaine behavioral effects in rats. *PLoS One* 8:e81018. 10.1371/journal.pone.0081018 24260531 PMC3832370

[B21] Kimura M. Tohya K. Kuroiwa K. Oda H. Gorawski E. C. Zhong X. H. (1992). Electron microscopical and immunohistochemical studies on the induction of “Qi” employing needling manipulation. *Am. J. Chin. Med.* 20 25–35. 10.1142/S0192415X92000047 1605128

[B22] Kwon S. Lee Y. Park H.-J. Hahm D.-H. (2017). Coarse needle surface potentiates analgesic effect elicited by acupuncture with twirling manipulation in rats with nociceptive pain. *BMC Complement. Altern. Med.* 17:1. 10.1186/s12906-016-1505-2 28049463 PMC5209881

[B23] Kwon Y. D. Pittler M. H. Ernst E. (2006). Acupuncture for peripheral joint osteoarthritis: a systematic review and meta-analysis. *Rheumatology* 45 1331–1337. 10.1093/rheumatology/kel207 16936326

[B24] Langevin H. M. Bouffard N. A. Badger G. J. Churchill D. L. Howe A. K. (2006). Subcutaneous tissue fibroblast cytoskeletal remodeling induced by acupuncture: evidence for a mechanotransduction-based mechanism. *J. Cell. Physiol.* 207 767–774. 10.1002/jcp.20623 16511830

[B25] Langevin H. M. Bouffard N. A. Churchill D. L. Badger G. J. (2007). Connective tissue fibroblast response to acupuncture: dose-dependent effect of bidirectional needle rotation. *J. Altern. Complement. Med.* 13 355–360. 10.1089/acm.2007.6351 17480137 PMC3065718

[B26] Langevin H. M. Bouffard N. A. Fox J. R. Palmer B. M. Wu J. Iatridis J. C. (2011). Fibroblast cytoskeletal remodeling contributes to connective tissue tension. *J. Cell. Physiol.* 226 1166–1175. 10.1002/jcp.22442 20945345 PMC3053527

[B27] Langevin H. M. Churchill D. L. Cipolla M. J. (2001a). Mechanical signaling through connective tissue: a mechanism for the therapeutic effect of acupuncture. *FASEB J.* 15 2275–2282. 10.1096/fj.01-0015hyp 11641255

[B28] Langevin H. M. Churchill D. L. Fox J. R. Badger G. J. Garra B. S. Krag M. H. (2001b). Biomechanical response to acupuncture needling in humans. *J. Appl. Physiol.* 91 2471–2478. 10.1152/jappl.2001.91.6.2471 11717207

[B29] Langevin H. M. Churchill D. L. Wu J. Badger G. J. Yandow J. A. Fox J. R. (2002). Evidence of connective tissue involvement in acupuncture. *FASEB J.* 16 872–874. 10.1096/fj.01-0925fje 11967233

[B30] Lao L. (1996). Acupuncture techniques and devices. *J. Altern. Complement. Med.* 2 23–25. 10.1089/acm.1996.2.23 9395637

[B31] Leake R. Broderick J. E. (1999). Treatment efficacy of acupuncture: a review of the research literature. *Integr. Med.* 1 107–115. 10.1016/S1096-2190(98)00033-X

[B32] Lee B. R. Kim H.-R. Choi E.-S. Cho J.-H. Kim N.-J. Kim J.-H. (2017). Enhanced therapeutic treatment of colorectal cancer using surface-modified nanoporous acupuncture needles. *Sci. Rep.* 7:12900. 10.1038/s41598-017-11213-0 29018212 PMC5635022

[B33] Lee J. H. Choi T. Y. Lee M. S. Lee H. Shin B. C. Lee H. (2013). Acupuncture for acute low back pain: a systematic review. *Clin. J. Pain* 29 172–185. 10.1097/AJP.0b013e31824909f9 23269281

[B34] Li W. M. Cui K. M. Li N. Gu Q. B. Schwarz W. Ding G. H. (2005). Analgesic effect of electroacupuncture on complete Freund’s adjuvant-induced inflammatory pain in mice: a model of antipain treatment by acupuncture in mice. *Jpn. J. Physiol.* 55 339–344. 10.2170/jjphysiol.RP001505 16356296

[B35] Liao H. Y. Hsieh C. L. Huang C. P. Lin Y. W. (2017). Electroacupuncture attenuates CFA-induced inflammatory pain by suppressing Nav1.8 through S100B, TRPV1, opioid and adenosine pathways in mice. *Sci. Rep.* 7:42531. 10.1038/srep42531 28211895 PMC5304170

[B36] Lim S. (2010). WHO standard acupuncture point locations. *Evid. Based Complement. Alternat. Med.* 7 167–168. 10.1093/ecam/nep006 19204011 PMC2862941

[B37] Lin J. G. Chen W. L. (2008). Acupuncture analgesia: a review of its mechanisms of actions. *Am. J. Chin. Med.* 36 635–645. 18711761 10.1142/S0192415X08006107

[B38] Lin M. T. Liu G. G. Soong J. J. Chern Y. F. Wu K. M. (1979). Effects of stimulation of acupuncture loci Ta-Chuei (Go-14), Nei-Kuan (EH-6) and Tsu-San-Li (St-36) on thermoregulatory function of normal adults. *Am. J. Chin. Med.* 7 324–332. 543487 10.1142/s0192415x79000295

[B39] Lu K. W. Hsu C. K. Hsieh C. L. Yang J. Lin Y. W. (2016). Probing the effects and mechanisms of electroacupuncture at ipsilateral or contralateral ST36–ST37 acupoints on CFA-induced inflammatory pain. *Sci. Rep.* 6:22123. 10.1038/srep22123 26906464 PMC4764889

[B40] Macdonald A. J. Macrae K. D. Master B. R. Rubin A. P. (1983). Superficial acupuncture in the relief of chronic low back pain. *Ann. R. Coll. Surg. Engl.* 65 44–46. 6218776 PMC2494194

[B41] Manheimer E. White A. Berman B. Forys K. Ernst E. (2005). Meta-analysis: acupuncture for low back pain. *Ann. Intern. Med.* 142 651–653. 10.7326/0003-4819-142-8-200504190-00014 15838072

[B42] Meridians and Acupoints Compilation Committee of Korean Oriental Medical Colleges (2015). *Principles of Meridians & Acupoints: a Guidebook for College Students*. Daejeon: Jongnyeonamu Publishing Co.

[B43] Park J. J. Akazawa M. Ahn J. Beckman-Harned S. Lin F. C. Lee K. (2011). Acupuncture sensation during ultrasound guided acupuncture needling. *Acupunct. Med.* 29 257–265. 10.1136/aim.2010.003616 21642648 PMC4196666

[B44] Park J. Y. Park J. Jeon S. Doo A. R. Kim S. N. Lee H. (2014). From peripheral to central: the role of ERK signaling pathway in acupuncture analgesia. *J. Pain* 15 535–549. 10.1016/j.jpain.2014.01.498 24524846 PMC4196675

[B45] Paulose M. Shankar K. Yoriya S. Prakasam H. E. Varghese O. K. Mor G. K. (2006). Anodic growth of highly ordered TiO 2 nanotube arrays to 134 μm in length. *J. Phys. Chem. B* 110 16179–16184. 10.1021/jp064020k 16913737

[B46] Shen E. Wu W. Y. Du H. J. Wei J. Y. Zhu D. X. (1973). Electromyographic activity produced locally by acupuncture manipulation. *Chin. Med. J.* 9 532–535.

[B47] Shi Y. Qi L. Wang J. Xu M. S. Zhang D. Wu L. Y. (2011). Moxibustion activates mast cell degranulation at the ST25 in rats with colitis. *World J. Gastroenterol.* 17 3733–3738. 10.3748/wjg.v17.i32.3733 21990955 PMC3181459

[B48] Shu Q. Chen L. He W. Zhou H. Liang F. (2016). A review of inflammatory signaling pathway regulated by acupuncture. *World J. Acupunct. Moxibust.* 26 63–69. 10.1016/S1003-5257(17)30013-2

[B49] Sorcar S. Grimes C. A. In S. I. (2018). The Biocompatibility of nanoporous acupuncture needles. *J. Acupunct. Meridian Stud.* 11 107–115. 10.1016/j.jams.2018.03.004 29635041

[B50] Stux G. Berman B. Pomeranz B. (2003). *Basics of Acupuncture*, 5th Edn. Berlin: Springer.

[B51] Vickers A. J. Cronin A. M. Maschino A. C. Lewith G. MacPherson H. Foster N. E. (2012). Acupuncture for chronic pain: individual patient data meta-analysis. *Arch. Intern. Med.* 172 1444–1453. 10.1001/archinternmed.2012.3654 22965186 PMC3658605

[B52] Wang S. M. Kain Z. N. White P. (2008). Acupuncture analgesia: I. The scientific basis. *Anesth. Analg.* 106 602–610. 10.1213/01.ane.0000277493.42335.7b 18227322

[B53] White A. Ernst E. (2004). A brief history of acupuncture. *Rheumatology* 43 662–663. 10.1093/rheumatology/keg005 15103027

[B54] Wu S. Y. Chen W. H. Hsieh C. L. Lin Y. W. (2014). Abundant expression and functional participation of TRPV1 at Zusanli acupoint (ST36) in mice: mechanosensitive TRPV1 as an “acupuncture-responding channel. *BMC Complement. Alternat. Med.* 14:96. 10.1186/1472-6882-14-96 24612851 PMC3984709

[B55] Zhang D. Ding G. H. Shen X. Y. Yao W. Zhang Z. Y. Zhang Y. Q. (2007). Influence of mast cell function on the analgesic effect of acupuncture of “Zusanli” (ST 36) in rats. *Zhen Ci Yan Jiu* 32 147–152. 17691569

[B56] Zhang X. Park H. J. Lee H. (2015). Do acupuncture needle size and needling depth matter? A laser Doppler imaging study. *Integr. Med. Res.* 4 66–67. 10.1016/j.imr.2015.04.07528664112

[B57] Zhao Z. Q. (2008). Neural mechanism underlying acupuncture analgesia. *Prog. Neurobiol.* 85 355–375. 10.1016/j.pneurobio.2008.05.004 18582529

[B58] Zhu L. Ma Y. Ye S. Shu Z. (2018). Acupuncture for diarrhoea-predominant irritable bowel syndrome: a network meta-analysis. *Evid. Based Complement. Alternat. Med.* 2018 1–12. 10.1155/2018/2890465 29977312 PMC5994265

[B59] Zhu X. Liu Z. Qin Y. Niu W. Wang Q. Li L. (2018). Analgesic effects of electroacupuncture at St25 and Cv12 in a rat model of postinflammatory irritable bowel syndrome visceral pain. *Acupunct. Med.* 36 240–246. 10.1136/acupmed-2016-011320 29720377

[B60] Zylka M. J. (2010). Needling adenosine receptors for pain relief. *Nat. Neurosci.* 13 783–784. 10.1038/nn0710-783 20581811

